# Zinc homeostasis in *Pseudomonas*

**DOI:** 10.1007/s10534-022-00475-5

**Published:** 2022-12-06

**Authors:** Verena Ducret, Diego Gonzalez, Karl Perron

**Affiliations:** 1 Microbiology Unit, Department of Plant Sciences, University of Geneva, Quai Ernest-Ansermet 30, 1211 Geneva 4, Switzerland; 2 Laboratory of Microbiology, Institute of Biology, Faculty of Sciences, University of Neuchâtel, Rue Emile-Argand 11, 2000 Neuchâtel, Switzerland; 3 Section of Pharmaceutical Sciences, University of Geneva, Rue Michel-Servet 1, 1205 Geneva, Switzerland

**Keywords:** Metal transporters, Zinc homeostasis, Pseudomonas, RND, CzcR, CzcS, CzcCBA, CadR, Zur, ZnuABC, Pseudopaline, P-type ATPase, CDF, ABC-transporters

## Abstract

In the genus *Pseudomonas*, zinc homeostasis is mediated by a complete set of import and export systems, whose expression is precisely controlled by three transcriptional regulators: Zur, CzcR and CadR. In this review, we describe in detail our current knowledge of these systems, their regulation, and the biological significance of zinc homeostasis, taking *Pseudomonas aeruginosa* as our paradigm. Moreover, significant parts of this overview are dedicated to highlight interactions and cross-regulations between zinc and copper import/export systems, and to shed light, through a review of the literature and comparative genomics, on differences in gene complement and function across the whole *Pseudomonas* genus. The impact and importance of zinc homeostasis in *Pseudomonas* and beyond will be discussed throughout this review.

**Graphical abstract:**

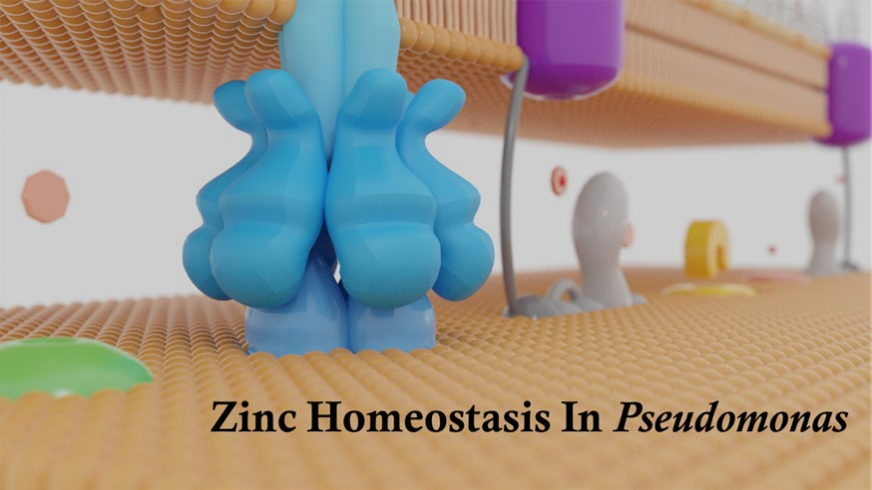

## Introduction

Zinc, which occurs as the divalent Zn^2+^ inside cells, is a trace element essential for life. It is a cofactor of many enzymes and stabilizes the structure of even more proteins, including transcription factors. In prokaryotes, zinc binds to 5 to 6% of proteins, playing both structural and functional roles (Chandrangsu et al. 2017). However, when present in excess, zinc becomes toxic mainly because it binds to metalloproteins normally associated with other metals from the Irving-Williams series and compromises their function: this phenomenon is called mismetallation (Foster et al. 2014). As a consequence, intracellular zinc concentrations have to be finely regulated at all times. This is done, in all living beings, through zinc homeostasis systems which control the uptake and release of the metal and maintain adequate cellular zinc concentrations. In bacteria, the cytoplasmic level of zinc stabilizes between 10^–3^ and 10^–4^ M, making it the second most abundant metal after iron; however, because zinc is largely complexed by its ligands, the free ions concentration is very low, ranging from 10^–12^ to 10^–14^ M (Chandrangsu et al. 2017).

Because bacteria are crippled by either lack or excess of zinc, many eukaryotes have evolved means to manipulate local zinc concentrations to counter bacterial infections. In mammals, zinc, like other essential metals such as iron and manganese, is sequestered from the serum and interstitial liquid to block pathogen development in a process called *nutritional immunity* (Kehl-Fie and Skaar 2010). Conversely, in some compartments of eukaryotic cells, zinc concentrations can reach inhibitory or even toxic levels (Djoko et al. 2015). In mammalian phagocytic cells, toxic concentrations of zinc are transferred from the cytoplasm to the phagosomes containing engulfed bacteria through the ZnT transporter family (Eide 2006; Huang and Tepaamorndech 2013). In the amoeba *Dictyostelium discoideum*, zincosomes, a particular type of endosome containing high levels of zinc, fuse with the phagosome to neutralize bacteria. The intracellular survival of bacteria therefore correlates with their capacity to export zinc: for instance, an *Escherichia coli* strain deficient in its primary zinc efflux system (ZntA) has been shown to be killed faster than the wild type in *D. discoideum* (Barisch et al. 2018). Plants also protect themselves against herbivores and phytopathogens by accumulating zinc in their tissues. This is well-illustrated in members of the *Brassicaceae*, such as *Noccaea caerulescens*, which can accumulate zinc up to 14 g/kg of dry weight. Such a concentration contributes to inhibit the growth of most pathogens. Typically, the metals are complexed by specialized molecules present in epidermal vacuoles and released during the destruction of these cells by the pathogen (Broadley et al. 2007). As a consequence, zinc plays a major role in host–pathogen interactions across the eukaryotic world (Hood and Skaar 2012; Lopez and Skaar 2018).

Some bacteria, however, have evolved efficient strategies to deal with extreme zinc concentrations and strong zinc fluctuations. Such bacteria have often been isolated from soil, which offers a wide range of zinc availability. For example, sandy soils in central Africa and southern Asia are severely deficient in zinc and thus force endemic plants and microorganisms to use strategies in order to recover the little metal available (Alloway 2009). Conversely, more and more sites contaminated with heavy metals, including zinc, are enriched in microorganisms which are highly metal-resistant (Diaz-Ravina and Baath 1996; Moffett et al. 2003).

Bacteria of the genus *Pseudomonas* are particularly robust when it comes to withstand extreme concentrations of zinc and other metals. They possess a complete arsenal of efflux systems that actively expel cytoplasmic or periplasmic metals out of the cell. Some species are also well-armed to recover metals and thrive under zinc-limited conditions. It is therefore not surprising to find among *Pseudomonas* species some major plant and animal pathogens able to withstand nutritional immunity and to evade phagocytosis. This makes the study of zinc homeostasis in these organisms of dual interest, both environmental and clinical.

*Pseudomonas aeruginosa* is a major opportunistic pathogen listed in 2017 as priority 1 for the discovery of antibacterial treatments according to the WHO (Tacconelli et al. 2018) WHO. Its ability to colonize its host heavily relies on its quick and effective responses to extreme zinc fluctuations. Accordingly, *P. aeruginosa* encodes versions of most zinc transport systems described in Gram-negative bacteria. Some of these zinc transporters have been shown to directly modulate virulence and antibiotic resistance (Perron et al. 2004; Dieppois et al. 2012; Ellison et al. 2013; D'Orazio et al. 2015). In addition, when exposed to Zn excess, *P. aeruginosa* becomes more virulent, causing gastrointestinal dysfunction and illness in a mouse model (Wu et al. 2021). For all these reasons, zinc homeostasis has been extensively studied in this species. While *P. aeruginosa* remains our the central reference model, the present review will broaden its scope to cover zinc homeostasis in the entire *Pseudomonas* genus.

## ZINC uptake

### From outside to the periplasm

**Fig. 1:**
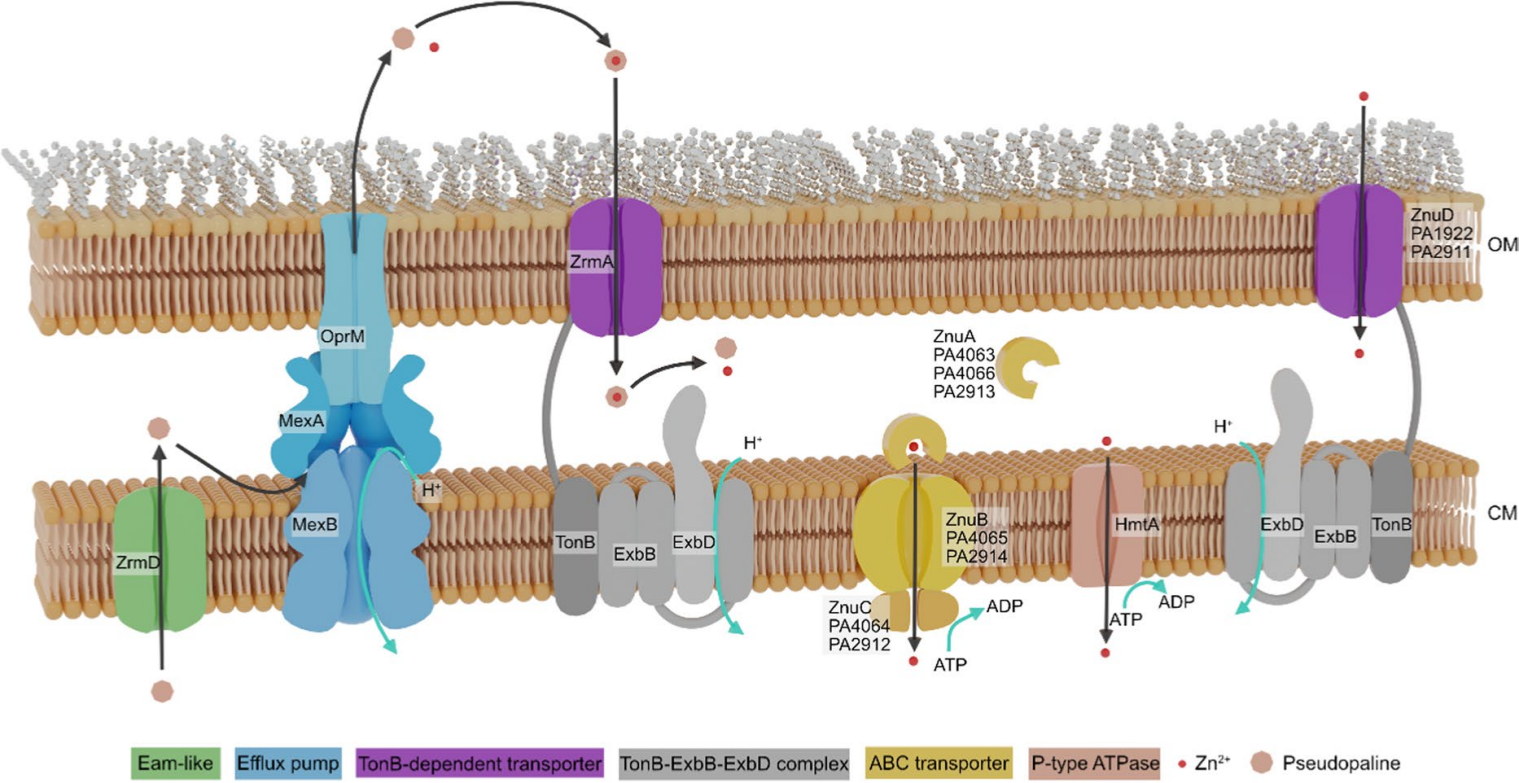
Schematic representation of transporter families involved in zinc uptake in *P. aeruginosa.* The gene name or ID in the reference genome of *P. aeruginosa* PAO1 (PA number) are indicated in the figure; the name of the transporter family is indicated below the figure with the corresponding color. *OM* outer membrane; *CM* cytoplasmic membrane

When available in the environment, elemental zinc is able to cross the outer membrane by passive or facilitated diffusion using ion channels (Cerasi et al. 2013). Active acquisition systems however are required when very low concentrations of the trace element are present in the surrounding environment. Active transport of zinc through outer membrane channels is driven by TonB-ExbB-ExbD complexes sitting in the cytoplasmic membrane; energy is provided through the H^+^ gradient and relayed to the outer membrane channel by the proline-rich spacer of TonB proteins (Stork et al. 2010; Krewulak and Vogel 2011), (Fig. 1). Four TonB-dependent receptors are involved in zinc import in *P. aeruginosa* (Pederick et al. 2015). ZnuD, for zinc uptake component **D**, and PA1922 appear to be responsible for the import of free Zn^2+^, while ZrmA (CntO), which is part of the pseudopaline system described below, and PA2911 seem to be specific for a chelated form of the metal.

### From the periplasm to the cytoplasm

In prokaryotes, the ATP-binding cassette (ABC-)permease ZnuABC is the most common mechanism allowing zinc to cross the cytoplasmic membrane (Fig. 1). First identified in *E. coli* (Patzer and Hantke 1998), this transport system is, based on the number of transmembrane helices of ZnuA a member of the type-I ABC transporter family (Davies et al. 2021). The transport of Zn^2+^ involves a periplasmic solute-binding protein (SBP), ZnuA, which captures the zinc ion and delivers it to the ZnuB transmembrane channel; the energy required for the translocation is provided by the ATPase ZnuC. The system operates according to a ZnuAB_2_C_2_ stoichiometry. The genes coding for the ZnuA, ZnuB and ZnuC proteins show 60%, 60% and 61% identity with their *P. aeruginosa* counterparts (Ellison et al. 2013).

**Fig. 2:**
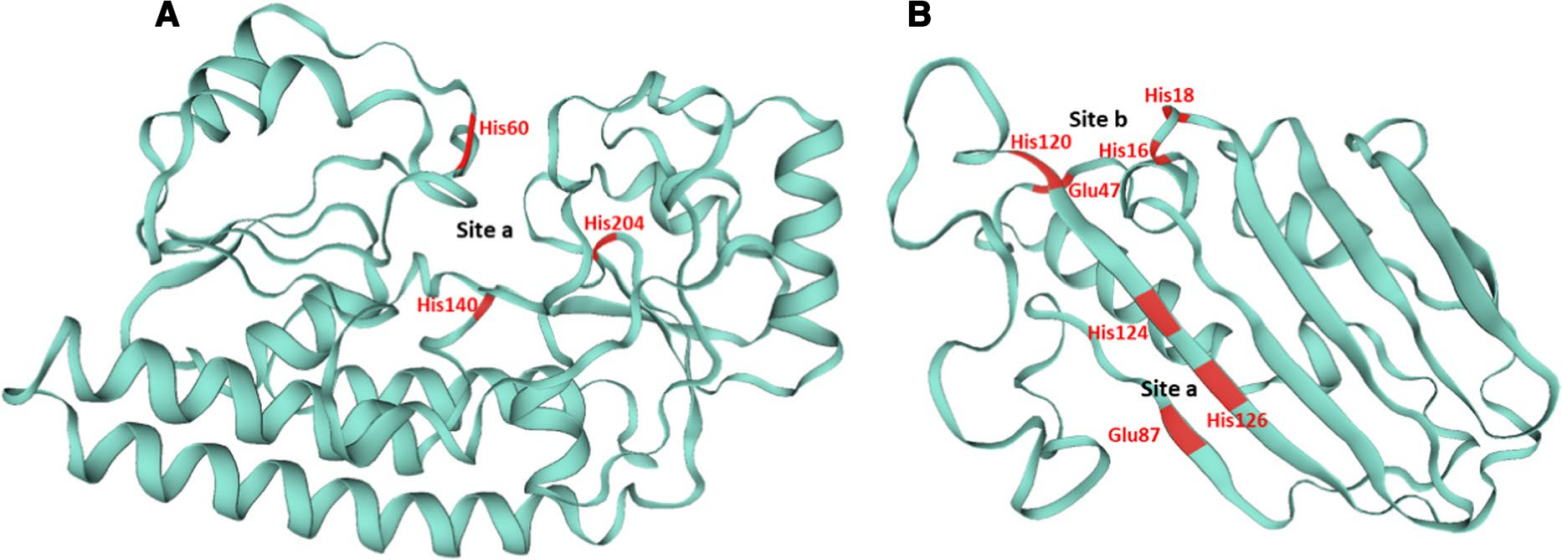
Cartoon representation of two *P. aeruginosa* SBPs.** A** ZnuA shows a typical cluster A-I fold. The unique zinc binding site (“Site a”) is indicated and the predicted Zn^2+^ binding residues are specified in red (Pederick et al. 2015).** B** PA4063 structure showing two Zn^2+^ binding sites (“Site a” and “Site b”). Residues involved in zinc coordination are indicated in red (Fiorillo et al. 2021). The two structures were determined using SWISS-MODEL (Waterhouse et al. 2018)

In *P. aeruginosa*, ZnuA harbors a histidine-rich region characteristic of Zn^2+^ SBP, where residues H60, H140 and H204 appear to be responsible for binding to a Zn^2+^ ion (*Site a* in Fig. 2A). An in silico reconstitution of the structure, shown in Fig. 2, reveals the typical A-I fold cluster resulting in a rigid structure (Pederick et al. 2015; Fukamizo et al. 2019). SBP metal specificity is sometimes difficult to determine. For instance, the *P. protegens* Pf-5 ABC transporter PFL_0643-0645 is annotated as a FeCT family iron-chelated transport system. However, the PFL_0644 SBP is overexpressed under zinc-limiting conditions and a Zur-box has been predicted on its promoter (Lim et al, 2013). Moreover, no substantial change in transcription has been observed under iron starvation versus iron excess conditions (Lim et al. 2012). Finally, considering that PA4045 (BtuF) and PA2913, the closest orthologues of PFL_0644 in *P. aeruginosa* (according to Pseudomonas.com)*,* are predicted to be a chelated cobalt (vitamin B12) and a chelated zinc transporter respectively (Borths et al. 2002; Pederick et al. 2015), we propose that PFL_0643-0645 might be an ABC transporter specific for complexed forms of zinc or cobalt. In *P. putida*, two ZnuABC systems are encoded in different operons, one of which may also be involved in the import of manganese (Canovas et al. 2003).

Besides ZnuABC, *P. aeruginosa* encodes two additional, poorly characterized, zinc ABC transporters (Pederick et al. 2015). The PA2914 system uses PA2913 as SBP, which falls in cluster A-II and is better suited to the transport of chelated ions (Pederick et al. 2015; Fukamizo et al. 2019). A recent study showed that, under conditions of zinc scarcity, the PA4063-PA4066 system was the ABC transporter with highest expression (Ducret et al. 2021). Intriguingly, this system has two probable SBPs: PA4063 and PA4066. Although these proteins exhibit a signal sequence that localizes them in the periplasmic space, they do not have a recognizable SBP structure. Moreover, only PA4063 contains a histidine stretch and therefore would be able to bind zinc (Pederick et al. 2015). This was recently confirmed by its crystallographic characterization that reveals two low-affinity zinc binding sites (Fig. 2B, (Fiorillo et al. 2021)). Additional experiments are needed to investigate the relative importance of these three systems in case of Zn deficiency.

Another type of system involved in zinc uptake is the P-type ATPase, a superfamily of transporters that translocate transition metals across the cytoplasmic membrane by hydrolyzing ATP. These systems usually act as efflux pumps. Nevertheless, in *P. aeruginosa*, overexpression of the P-type ATPase HmtA (for heavy metal transporter A) induces a hypersensitivity to zinc and copper and increased intracellular concentration of these metals, suggesting that the metal might be transported from the periplasm to the cytoplasm (Lewinson et al. 2009). Further analyses are needed to confirm this unusual characteristic. Moreover, it has been shown to be induced in a *ΔznuA* mutant (Pederick et al. 2015), suggesting an involvement in the zinc uptake pathway. Surprisingly, this transporter is the only known import system which is not repressed by zinc excess (Ducret et al. 2021). Thus, it would act as a safety mechanism in the event of sudden zinc deficiency or could simply be expressed for its role in copper entry.

Finally, zinc uptake in *P. aeruginosa* can also be driven by the ZrmABCD system, which involves a staphylopine-like molecule called pseudopaline (Mastropasqua et al. 2017). This zincophore is synthesized in the cytoplasm by the ZrmB (CntL) and ZrmC (CntM) enzymes; it crosses the cytoplasmic membrane via the EAM-like transporter ZrmD (CntI) (McFarlane and Lamb 2017) and is exported to the extracellular space through the MexAB-OprM efflux pump (Fig. 1) (Gomez et al. 2021). Pseudopaline bound to extracellular zinc or nickel ions is then reinternalized into the periplasmic space by the TonB-dependent transporter ZrmA (CntO) (Lhospice et al. 2017; Mastropasqua et al. 2017). The way the complex transits back to the cytoplasm remains unknown for the moment; but recent data suggests that pseudopaline is modified in the periplasm and probably releases the metal in this compartment (Gomez et al. 2021).

Nicotianamine-like metallophores like pseudopaline probably appeared early in evolution (Laffont and Arnoux 2020). Under the anoxic conditions of primitive Earth, which allowed for high concentrations of zinc, manganese and phosphate ions in solution, metal chelation could have helped with metal resistance (Laffont and Arnoux 2020). To cope with metal deprivation resulting from later oxidation events, metallophores could have then neofunctionalized in metal acquisition. They might only have been preserved in microorganisms evolving in metal-scarce conditions, as found within eukaryotic hosts or in sandy soils. Interestingly, the pseudopaline system is almost exclusively conserved in *P. aeruginosa;* typical environmental species like *P. fluorescens* or *P. stutzeri* do not present any close homolog of the pseudopaline synthesis genes (see below).

### Zinc recycling

Zinc is used as a cofactor in 5 to 6% of the bacterial proteome (Andreini et al. 2006). This represents a significant stock of metal that can be mobilized by the cell under limiting conditions. “Zinc recycling,” also referred to as “zinc sparing” or “zinc economy” (Merchant and Helmann 2012), is a process proposed to increase intracellular zinc availability for essential functions: it involves replacing some zinc-binding proteins by paralogs which do not require zinc. These paralogs have lost their cysteine-containing Zn^2+^-binding domain; they are therefore referred to C − versions by contrast with the zinc-dependent C + family of proteins. C − paralogs are usually repressed under zinc-replete conditions but are expressed and can functionally replace their C + paralogs during metal starvation. In *P. aeruginosa*, C − paralogs are often grouped in operons. For instance, the *PA3601-PA3600* transcription unit encodes for the C − ribosomal proteins RpmE2 and RpmJ2 respectively and the *PA5536-PA5534* unit encodes for the transcriptional factor *dksA2*, involved in the stringent response, and genes belonging to the COG0523 family. Members of this protein family have a number of functions, including cobalamin biosynthesis, GTPases or metallochaperones and those aimed at sparing inadequate cellular zinc distribution (Crouzet et al. 1991; Haas et al. 2009; Jordan et al. 2019). For instance, the COG0523 G3E protein is a GTP-dependent metallochaperone involved in the maturation of metal-containing enzymes that are typically upregulated under conditions of zinc starvation (Haas et al. 2009; Capdevila et al. 2017). Another set of genes involved in the production of zinc-independent paralog enzymes, *PA5539-PA5541*, encodes for the GTP hydrolase FolE2 (PA5539), a putative γ-carbonic dehydratase (PA5540), and the dihydroorotase PyrC2 (PA5541) (Pederick et al. 2015). Similar clusters encoding non-zinc-requiring paralogs have been identified in *P. protegens* (Lim et al. 2013).

Replacement of C + by C − paralogs has been proposed to foster metal release when zinc availability becomes critical (Akanuma et al. 2006). This hypothesis is however controversial. For example, in *E. coli*, the ratio between RpmE and RpmJ and their respective C − paralogs has a direct impact on translation, suggesting a divergent role. Moreover, RpmE2 and RpmJ2 appeared to be expressed in late exponential growth phase, even in the presence of zinc (Lilleorg et al. 2020). This was also observed in *P. protegens* Pf-5, in which qRT-PCR revealed that expression of *rpmE* and *rpmJ* did not change under zinc-limitation (Lim et al. 2013). Similarly, in *P. aeruginosa*, several C + and C − paralogs are co-expressed even in metal-poor environments (Ducret et al. 2021). In this bacterium however, DksA2 and PyrC2 were clearly able to complement the deletion of their respective paralog and are therefore considered as backup copies induced in response to zinc deprivation (Brichta et al. 2004; Blaby-Haas et al. 2011).

## ZINC export

### Export machinery

**Fig. 3:**
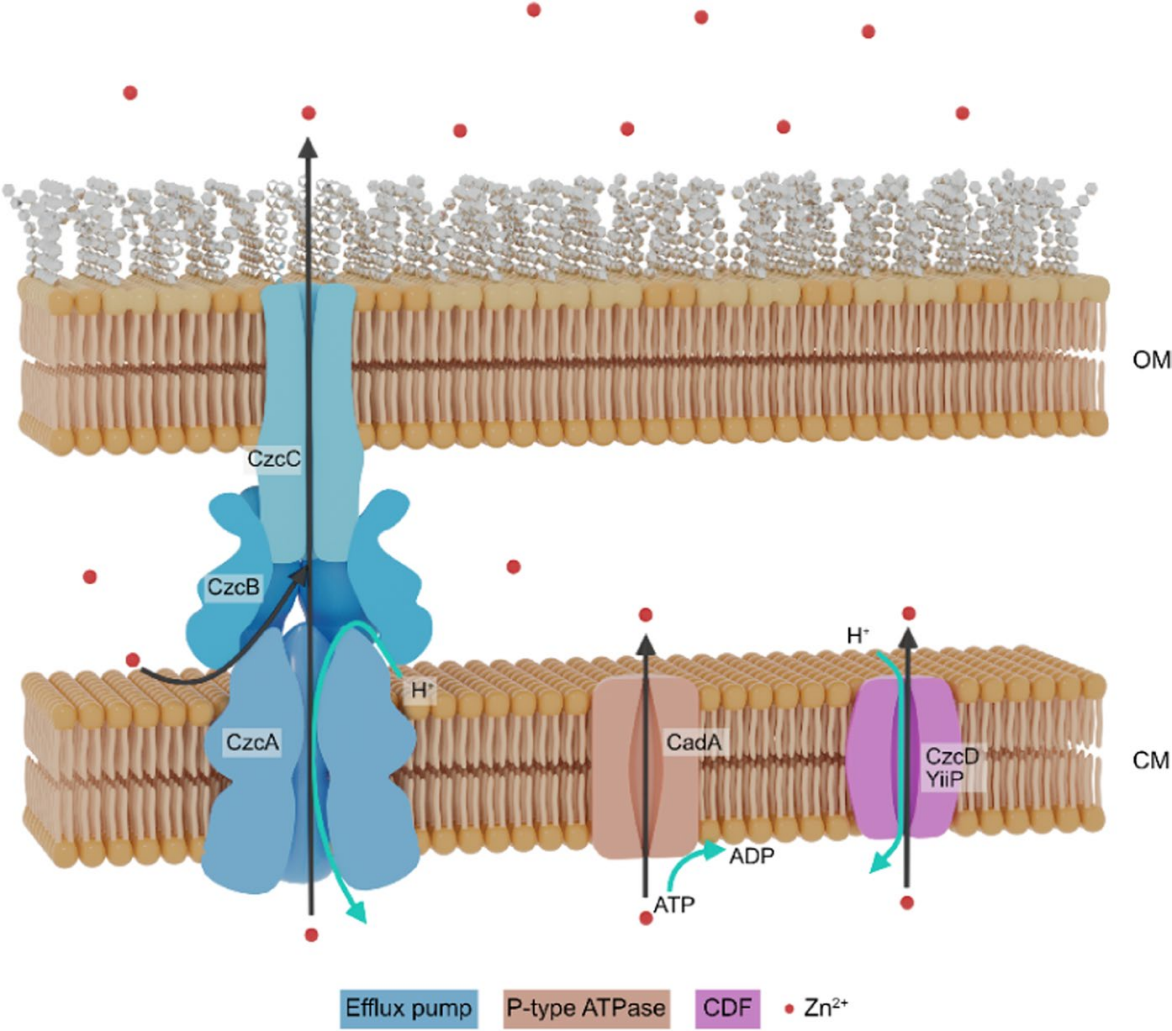
Schematic representation of transporter families involved in zinc export in *P. aeruginosa.* The gene name or ID in the reference genome of *P. aeruginosa* PAO1 (PA number) are indicated in the figure; the name of the transporter family is indicated below the figure with the corresponding color. *OM* outer membrane; *CM* cytoplasmic membrane

Three types of transporters are involved in zinc export (Fig. 3). The CzcCBA RND efflux pump is by far the most effective, according to the level of resistance it confers. This complex belongs to the Heavy Metal Efflux (HME) Resistance Nodulation Division (RND) superfamily (Mergeay et al. 1985; Hassan et al. 1999); it was initially discovered and then studied in depth in *Cupriavidus metallidurans*, a bacterium known for its high resistance to heavy metals. CzcCBA is a tripartite complex with a CzC_3_B_6_A_3_ stoichiometry (Fig. 3). CzcC makes a beta barrel channel across the outer membrane; the RND family protein CzcA spans the cytoplasmic membrane with twelve alpha helices and the membrane fusion protein CzcB forms a ring that stabilizes the contact between CzcA and CzcC. In the presence of zinc or cadmium excess, the CzcCBA machinery utilizes the proton motive force to actively expel zinc, cadmium, and cobalt from the cell (Goldberg et al. 1999). Two mechanisms of metal export have been proposed. In the first model, zinc transits trough the periplasm: it is transferred to the metal binding site of CzcA via CzcB and then expelled through CzcC. This model seems currently the most likely. The second route allows zinc to be pumped out of the cytoplasm and ejected directly in the extracellular medium without releasing it into the periplasm (Kim et al. 2011). In some *Pseudomonas* species, this system is supplemented by accessory proteins. In *P. stutzeri* RCH2 for example, an additional periplasmic protein CzcI may act as a metal ion chaperone, facilitating the expulsion of periplasmic zinc by delivering it to CzcB (Vaccaro et al. 2016). The potential cytoplasmic membrane CzcN in *P. putida* KT2440 is another example of CzcCBA partner (Canovas et al. 2003). Interestingly, the genome of this bacterium encodes a doublet of the *czcCBA* operon and five additional *czcA* genes although only the CzcA1 and CzcA2 are close enough to the *Cupriavidus* CzcA to confer zinc resistance (Canovas et al. 2003). Moreover, the second copy of the *czc* operon (*czc*2) appears to serve as a backup since it is expressed only in a *czc*1-deficient background (Mumm et al. 2016). In *C. metallidurans*, an accessory protein called CzcE is localized in the periplasm; it is induced by zinc, but yet, was only shown to play a role in copper homeostasis, binding two copper ions (I or II) as a dimer (Zoropogui et al. 2008). The protein encoded by *PA2807* in *P. aeruginosa* is a homolog of CzcE (Ducret et al. 2021), but is located in a copper cluster and co-transcribed with *ptrA* and *queF* (Quintana et al. 2017). This cluster is known to be part of the copper response two-component system CopRS regulon. Interestingly, this operon has also been shown to respond to zinc excess thanks to the two-component system CzcRS described below (Ducret et al. 2021). Therefore, induction of the *ptrA-PA2807-queF* operon occurs via two different two-component systems, depending on the inducing metal.

The Cation Diffusion Facilitator (CDF, Fig. 3) is the last known export system in *P. aeruginosa*. First described in 1997, CDFs were primarily thought to be involved in metal tolerance, functioning as proton antiporters to expel divalent cations across the cytoplasmic membrane (Paulsen and Saier 1997; Kolaj-Robin et al. 2015). Indeed, in some bacteria, CzcD and YiiP, which occur as homodimers or heterodimers, are key players in zinc homeostasis (Anton et al. 1999; Wei and Fu 2005). Structural analyses show that CDFs are composed of six transmembrane segments and a cytoplasmic C-terminal domain, important for transport regulation, but also for dimerization. Four zinc binding sites have been identified within a conserved “Site A” located in transmembrane helices 2 and 5, indispensable for transport (Paulsen and Saier 1997; Kolaj-Robin et al. 2015). In *P. aeruginosa*, CDFs are not essential for zinc resistance but may play a role in membrane integrity (Salusso and Raimunda 2017). In agreement with this, constitutive expression of CzcD is observed after addition of zinc, with only a weak and late induction (Ducret et al. 2020, 2021). Multiple copies of these transporters are found in the genomes of several *Pseudomonas* species. The presence of paralogs is likely the result of horizontal acquisitions or duplications and probably supports improved resistance to transition metals (Canovas et al. 2003; von Rozycki and Nies 2009).

### Entrap zinc to survive?

We cannot conclude without considering the metallothioneins, a family of small proteins involved in sequestering excess metals in the cytoplasm. The first of these proteins identified in bacteria, SmtA, was discovered in 1979 in the cyanobacterium *Synechococcus elongatus* as a significant player in zinc detoxification (Olafson et al. 1979). To date, very few of these proteins have been described. These cytoplasmic proteins, rich in cysteine residues, are capable of scavenging a broad spectrum of essential and non-essential metals, forming metal-thiolate clusters. Metallothioneins can thus finely buffer the cytoplasm (Chatterjee et al. 2020). In addition, the numerous cysteine residues confer better resistance against oxidative stress by scavenging reactive oxygen and nitrogen species (Spahl et al. 2003; Zeitoun-Ghandour et al. 2011). In *Pseudomonas* however, a role for these proteins in zinc homeostasis has not yet been demonstrated. Two metallothioneins have been described, one in *P. fluorescens Q2-87* and another in *P. aeruginosa*; however, both are expressed mainly in late stationary phase (after 24 to 48 h of growth) in planktonic cells or biofilms, and seem to provide a fitness benefit for long-term survival (Habjanic et al. 2018, 2020).

## Regulation of zinc homeostasis

### Regulation of zinc homeostasis under zinc limitation or sufficiency

**Fig. 4:**
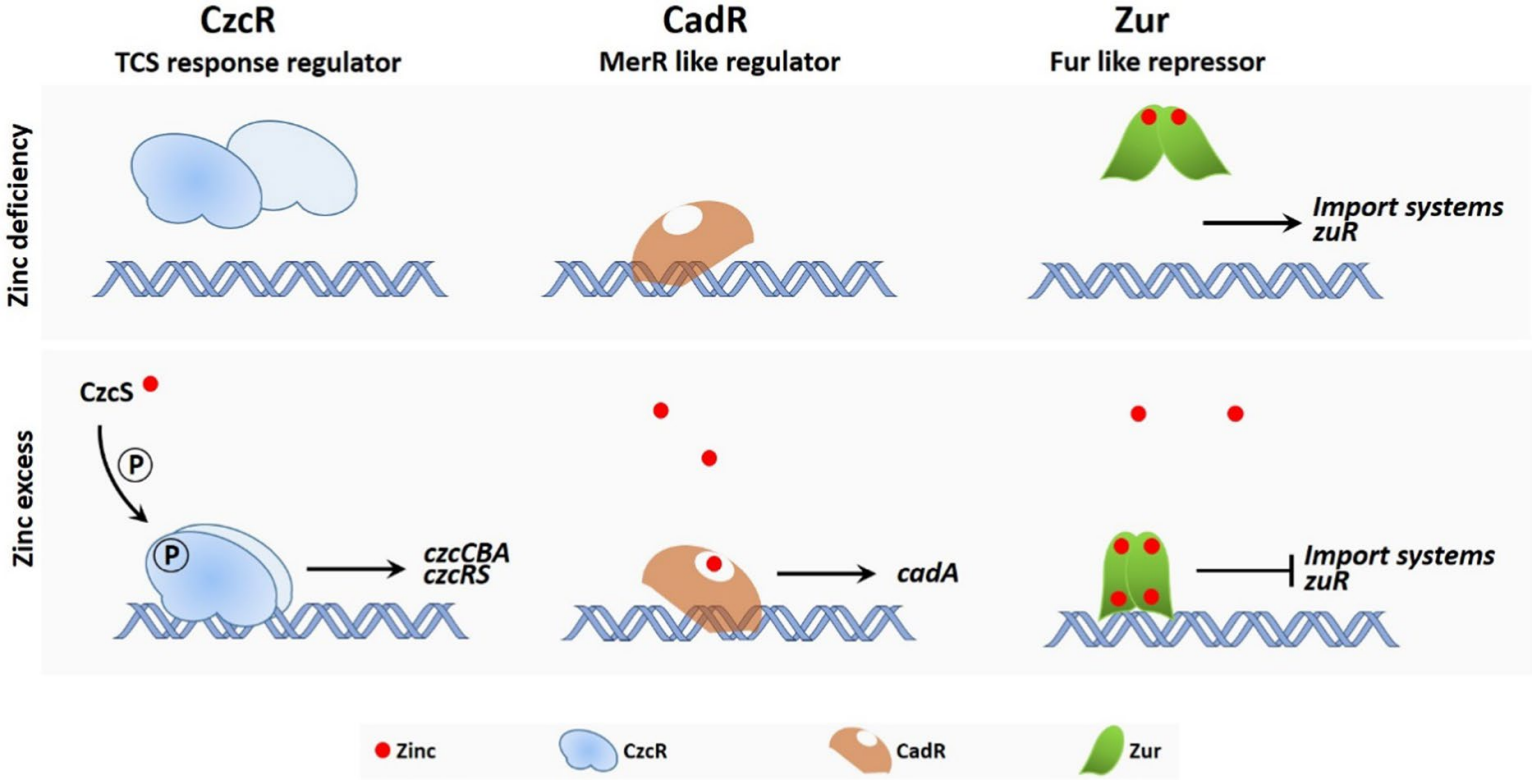
The three types of transcriptional regulators involved in zinc homeostasis in *Pseudomonas*. Left panel: CzcR (in blue) is part of the CzcRS TCS. Periplasmic Zn is detected by the sensor CzcS, which activates the CzcR regulator by phosphorylation. This induces CzcR dimerization and its binding to the promoters of *czcRS* and *czcCBA*, activating their transcription. Middle panel: CadR (in orange) is a member of the MerR-type family of regulators capable of binding to the promoter in the absence of Zn. The binding of Zn to CadR induces an allosteric change, activating the transcription of the P-type ATPase CadA. Right panel: At low cellular Zn concentration, the Zur protein (in green) is in a dimeric form with a ratio of one Zn atom per monomer and does not bind DNA. At high Zn concentration, Zur adopts a different conformation, each Zur monomer binding two Zn atoms, and becomes capable of binding DNA and repressing genes involved in Zn import

The expression of zinc uptake systems and C − paralog proteins is transcriptionally regulated by the Zinc Uptake Regulator, Zur (Fig. 4). This transcriptional regulator belongs to the FUR (Ferric Uptake Regulator) family (Fillat 2014) and is widely conserved in bacteria, including proteobacteria (Panina et al. 2003). Like other allosteric regulators, Zur changes its DNA binding characteristics upon binding free cytoplasmic zinc (Ellison et al. 2013; Gonzalez et al. 2019). It possesses a very strong affinity for Zn with a constant of 6.4 × 10^–13^ M (Osman et al. 2015) that enables a graded response based on the concentration of the metal, allowing for a hierarchical regulation of targets. In Apo-Zur, only the structural site, located in the C-terminal region (C-site), is generally bound by zinc; the protein then forms a dimer (Zur_2_:Zn_2_) unable to bind DNA. When zinc concentration increases, the regulator is found in an intermediate Zur_2_:Zn_3_ state, in which the regulatory M-site of only one Zur per dimer is occupied by zinc; this conformation allows the dimer to bind DNA with low affinity. At higher zinc concentrations, the two M-sites of the dimer are occupied by zinc and this Zur_2_:Zn_4_ conformation leads to strong DNA binding activity (Shin and Helmann 2016). When bound to DNA, Zur usually functions as a repressor preventing the RNA polymerase from initiating transcription. In *P. aeruginosa*, the Zur protein, formerly known as NP20, has been characterized (Ellison et al. 2013). The Zur DNA binding site (Zur box) is defined as a 17 bp palindromic motif that often overlaps with the − 10 of target promoters. With the exception of the *hmtA* gene, Zur was found to bind promoters of all genes involved in zinc uptake, but also promoters of C − paralog proteins, in presence of zinc excess (Ducret et al. 2021). In several bacteria, including *Xanthomonas campestris, Streptomyces coelicolor* and *Caulobacter crescentus,* Zur can also act as an activator of zinc efflux genes (Huang et al. 2008; Mazzon et al. 2014; Choi et al. 2017). The localization of the Zur boxes on the promoter and the ability of the protein to multimerize at high zinc concentrations seem to determine whether the regulator functions as a repressor or an activator. This highlights the contribution of Zur to the adaptability of bacteria under zinc scarcity or excess.

### Zinc homeostasis regulation under zinc excess

The high adaptability of the *Pseudomonas* genus is supported by numerous two-component systems (TCS) that allow a rapid and targeted response to environmental stimuli (Rodrigue et al. 2000). The CzcCBA efflux pump, for instance, is regulated by the CzcRS TCS (Fig. 4). CzcS is a cytoplasmic membrane-located sensor protein capable of detecting an excess of periplasmic cadmium or zinc, with an affinity of 1.7 × 10^–6^ M (Hassan et al. 1999; Perron et al. 2004; Wang et al. 2017). The ligand induces dimerization and autophosphorylation of the sensor on a conserved histidine residue. In this conformation, it activates the CzcR regulator by transferring its phosphoryl group to the regulatory aspartate residue. In turn, this protein directly activates both the transcription of the *czcCBA* operon and its own. The *N*-terminal periplasmic domain of the *P. aeruginosa* CzcS protein has been crystalized and its structure determined (Wang et al. 2017). Importantly, the structure shows that zinc is directly recognized by CzcS, without the need for additional periplasmic proteins. In *P. aeruginosa,* twelve response regulators of TCS might bind the intergenic region between *czcRS* and *czcCBA* forming CzcR as the “key downstream node of RR network” (Trouillon et al. 2021). Besides, CzcR exhibits a dual role: in addition to promoting zinc, cadmium, and cobalt resistance via the activation of the *czcCBA* efflux pump, it downregulates the expression of *oprD* by directly binding the promoter region*.* OprD is a porin involved in the entry of basic amino acids and carbapenem antibiotics (imipenem and meropenem), a very important class of antimicrobial compounds active against *Pseudomonas* (Dieppois et al. 2012). Its repression in the presence of zinc makes *P. aeruginosa* resistant to this family of antibiotics. Surprisingly, in *P. stutzeri* RCH2, CzcR has an opposite effect since it activates the expression of the OprD porin (Garber et al. 2018). Nevertheless, the biological reason for the zinc-dependent regulation of this porin remains a mystery. Many virulence factors are also controlled by CzcR, which can therefore be considered a global regulator connecting metal resistance with antibiotic resistance and virulence (Dieppois et al. 2012). Interestingly, some of these genes do not require zinc excess to be regulated, suggesting a basal CzcR activity. Recently, the CzcRS regulon was further expanded, since it appears that the zinc-mediated induction of *czcD*, along with that of *ptrA* and *czcE*, also hinges on this TCS (Ducret et al. 2021). Remarkably, two *czcRS* operons with an additional *czcR3* have been identified in *P. putida*, all induced by zinc; this highlights the aptitude for sensor proteins to phosphorylate at least one or two different CzcR (Mumm et al. 2016).

In *P. aeruginosa*, proper activation of CzcRS requires the CadA exporter, a P-type ATPase regulated by CadR (ZntR) (Fig. 4). A dual role has been attributed to this transcriptional regulator: while it clearly acts as an inducer in the presence of zinc, it also appears to exert slight negative control in conditions of metal limitation (Ducret et al. 2020). The *cadR* gene is located just upstream of *cadA*, but transcribed in the opposite direction. It belongs to the MerR-like family and should bind between the − 35 and − 10 of target promoters (Brown et al. 2003). Under starvation conditions, CadR bends the DNA around the operator and prevents the RNA polymerase from initiating transcription. At high zinc concentrations, an allosteric change in the regulator bound to the metal, with an affinity constant of 3.2 × 10^–12^ M (Osman et al. 2015), relieves the DNA and allows the RNAP to start transcription.

### The crossing pathways between zinc and copper homeostasis

The response to zinc is often intimately linked to the response to copper. In bacteria, the cross-resistance to zinc and copper could be linked to the presence of both metals in the phagosomes of macrophages or protozoa. Indeed, numerous examples of Zn-Cu coregulations have been described in both Gram-positive and Gram-negative bacteria. For instance in *Enterococcus faecalis,* a general transcriptional network responds to both metals (Latorre et al. 2015) and numerous efflux systems of *Acinetobacter baumanii* respond to both Zn and Cu (Hassan et al. 2017). In *P. aeruginosa*, the *czcE* and *ptrA* genes are located on a copper cluster. Deletion of these genes appears to increase copper and zinc sensitivity, definitively linking these proteins to the homeostasis of both metals (Elsen et al. 2011; Ducret et al. 2021). By contrast, CzcE does not affect copper tolerance in *P. putida*. In *P. aeruginosa*, copper also has a positive effect on zinc resistance. This increased resistance is the consequence of the direct interaction of CopR, the transcriptional regulator of the copper specific TCS, with the *czcR* promoter (Caille et al. 2007). In *P. stutzeri*, this interconnection goes further, since CzcR, CopR1 and CopR2 have almost the same gene targets. This might be explained by the presence of a very similar DNA binding site for the three regulators (Garber et al. 2018). Although a link exists between zinc and copper in different species, the nature of the interplay between the two can be very different. In *P. stuzeri*, for instance, zinc induces the expression of the copper responding TCS (CopRS) via CzcRS (Garber et al. 2018), unlike what has been shown in *P. aeruginosa* (Caille et al. 2007). Interestingly a zinc-copper overlap is also found under metal starvation conditions. For instance, HmtA imports exclusively zinc and copper, unlike the conventional P-type ATPases that are either specific for zinc-cadmium or for copper-silver (Lewinson et al. 2009). Another example is the cytoplasmic metallochaperone CopZ and the porin OprC, related to copper metabolism, that are highly downregulated when *P. protegens* Pf-5 is exposed to a limiting amount of zinc (Lim et al. 2013). Why resistance to Zn and Cu is co-regulated is unclear. However, the antibacterial effect of these metals and their co-localization in phagosomes strongly suggest that a coordinated response to Zn and Cu excess is advantageous (Djoko et al. 2015). Deciphering the molecular basis of this co-regulation would be key to improve our understanding of the role of metals in host–pathogen interactions.

### Zinc homeostasis beyond *P*. *aeruginosa*

The data available on zinc homeostasis systems in *P. aeruginosa* is disproportionate compared to other *Pseudomonas* species. Although we have mentioned the few experimental studies covering parts of the system in *P. protegens, P. putida, P. stutzeri,* and *P. syringae,* an overview for the entire genus is still missing. To start closing the gap, we have surveyed the proteomes of 1254 sequenced *Pseudomonas* strains, available on the NCBI database, including about 300 strains not belonging to the *aeruginosa* group, using bioinformatics. Briefly, we looked for the best reciprocal protein *blastp* (v. 2.8.1 +) hit (Altschul et al. 1990, 1997) of all proteins known to be involved in zinc homeostasis in *P. aeruginosa* PAO1 in our genome set, retaining only reciprocal hits with an e-value < 0.01 and a query coverage of at least 75% (with the exception of CzcB, which has an *N*-terminal extension in PAO1); the requirement for a reciprocal hit and high query coverage ensured that the evalues were in general very low and that the likelihood of false positives was minimized. Strains had been clustered into large phylogenetic groups (“*aeruginosa*”, “*chlororaphis-protegens*”, “*fluorescens*”, “*putida*”, “*stutzeri*” and “*syringae*”) based on a tree built from an alignment of concatenated conserved ribosomal proteins using *fasttree* (Price et al. 2010). Table 1A presents the percentage of strains within each group encoding at least one possible homolog for each protein; Table 1B gives the median of the *blast* best hit evalues within each group and highlights empty cells and potential false positives.

The P1B (or heavy metal transporting)-ATPase CadA (or ZntA) is a second system involved in zinc resistance in *P. aeruginosa* (Fig. 3). The presence of a long histidine-rich tail might suggest that this protein is involved in the transport of cadmium (Arguello 2003), with a possible partial function in zinc and lead export from the cytoplasm to the periplasm (Ducret et al. 2020). As the first export system to be induced during a metal boost, CadA rapidly removes zinc excess from the cytoplasm. In addition, the zinc transferred to the periplasmic compartment is thought to play a major role in *czcCBA* induction (Ducret et al. 2020). *P. putida* KT2440 contains two functional *cadA* genes, one in the same genetic context as in *P. aeruginosa*, the other one located in the middle of the *czc* locus (Canovas et al. 2003; Leedjarv et al. 2008). Surprisingly, CadA was shown to be poorly conserved across the *Pseudomonas stutzeri* species (Table 1).

**Table 1:**
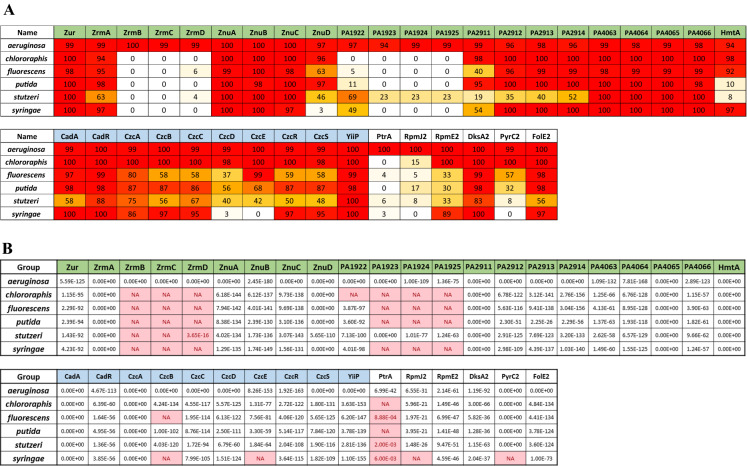
**A** Percentage of strains within major phylogenetic groups among *Pseudomonas* encoding at least one possible homolog of proteins involved in zinc homeostasis in *P. aeruginosa* PAO1. The red to orange to white gradient is proportional to the percentage, with red corresponding to 100%, orange to 50%, and white to 0%. **B** Median evalue of the best hits fore each protein within each group. Groups lacking a protein or presenting homologs with high evalues are highlighted in pink. Column name color code: Green: Zn uptake systems; blue: Zn export systems; white: C − paralog

Two features clearly put *P. aeruginosa* apart within the *Pseudomonas* genus when it comes to zinc import: 1. the pseudopaline system does not seem to be present in any other group (with the exception of homologs of ZrmA, which belongs to a widespread group of TonB transporters, difficult to discriminate on a bioinformatic basis); 2. the PA1922-25 system, which is suspected to encode an nonchelated-zinc import machinery, does not have orthologs in most other *Pseudomonas* species. This suggests that *P. aeruginosa* has invested more than other species into alternative zinc import systems, which presumably makes it better equipped to face nutritional immunity in mammalian plasma than purely environmental strains. Likewise, the C − proteins RpmJ2 and RpmE2 seem to be mostly restricted to the *aeruginosa* group (with the interesting exception of members of the *syringae* group, which have a RpmE2 ortholog), while PyrC2 and FolE2 homologs are found more widely in environmental *Pseudomonas* strains. We speculate that C- ribosomal proteins could be particularly advantageous for pathogens, like *P. aeruginosa* and *P. syringae,* for which high growth rates under limited zinc concentrations might be selective. On the other hand, the homologs of the three ABC transporters, ZnuABC, PA4063-66, and, to a lesser extent, PA2911-14, may be ancestral to the *Pseudomonas* genus and appear to be conserved independently from environment or lifestyle. Interestingly, the TonB transporters ZnuD, PA1922, and PA2911, which are responsible for the import of metallic zinc through the external membrane, do not seem to be as conserved as the cytoplasmic membrane components; different groups seem to have preferentially retained different paralogs (ZnuD and PA2911 are present in the majority of *putida* and *chlororaphis* strains, while PA1922 is absent; by contrast, PA1922 is frequent in the *stutzeri* group, where PA2911 is underrepresented). This suggests that there is some redundancy among TonB transporters involved in zinc import and that different patterns of gain and loss may have prevailed in different phylogenetic groups or environments. Among the proteins involved in zinc export, the most conserved in *Pseudomonas* strains is the CDF YiiP; an ortholog of CzcD is found in most *aeruginosa* and *chlororaphis* strains, but in less than half of the other strains. The efflux pump CzcCBA is usually found together with the CzcRS two component system; these systems are very conserved in all groups except *fluorescens* and *stutzeri*, where proper orthologs are missing in about 50% of strains. This relatively patchy distribution of export systems suggests that zinc excess might not be as strong a selective force in some natural environments as it is during pathogenesis in animals. Finally, we looked for Zur boxes in the promoter region of the different homologs we found (Table 2). This data supports a rather high conservation of the Zur-dependent regulation for *zur, zrmA, znuD, znuA, PA4063, rpmE2,* and *dksA2* (and following genes in the case of polycistronic RNAs); the regulation of *PA4063* and *dksA2* by Zur, however, seems to be lacking in specific groups (*putida* and *syringae*). This quite high stability of targets is consistent with the broad structural and functional conservation of Zur across Proteobacteria. The regulation by Zur of *PA1922* and *czcR* on the other hand seems unique to *P. aeruginosa.*

**Table 2:**
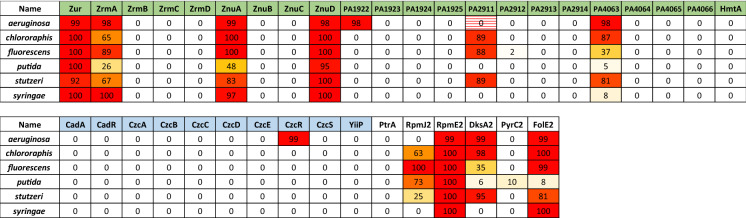
Percentage of *P. aeruginosa* PAO1 zinc-homeostasis homologs whose promoter region (150 bps upstream of the ATG) contains a Zur binding site (consensus: GWWAYWNNNTWHCR (Pederick et al. 2015)) within major phylogenetic groups among *Pseudomonas*. In *P. aeruginosa*, PA2911 is known to be regulated by Zur through a Zur-box located > 300bps upstream of the ATG, which was not picked up by the algorithm. Green: Zn uptake systems; blue: Zn export systems; white: C − paralogs

Overall, it seems that, apart from the exceptions mentioned earlier, the zinc homeostasis systems present in *P. aeruginosa* are mostly representative of the genus. There is, however, a notable tendency for strains in the *fluorescens* and the *stutzeri* groups to lack some systems widespread elsewhere. These environmental bacteria might be less exposed to zinc fluctuations or rely on promiscuous systems involved in the homeostasis of other metals, like copper or cadmium.

## Concluding remarks

The genus *Pseudomonas* includes bacteria with such versatility that they can be found in all environments, worldwide. All species in the genus possess a range of transporters and regulators supporting their survival and growth under both zinc scarcity and zinc excess. Although these systems are almost all represented within the genus, their number, their arrangement on the chromosome, and their effects on cell physiology seem to be species-specific. This is the case, for example, in zinc excess situations where the OprD porin is repressed in *P. aeruginosa* while it is overexpressed in *P. stuzeri* (Perron et al. 2004; Garber et al. 2018). The link between zinc and the expression of virulence factors in *P. aeruginosa* has stimulated the study of metal homeostasis in this pathogen. Unfortunately, the effects of the lack or excess of zinc in other species is poorly studied and deserves further investigations. With this in mind, future research in the field should focus on:

i. The interconnection between the various transcriptional regulators involved in both zinc and copper resistance, and both the import and export of these metals. For example, the localization of Zur, the repressor of import systems, on the promoter of *czcCBA* involved in Zn export confers an additional level of complexity, which still remains to be deciphered.
ii. The importance of Zn homeostasis should also be studied in plant pathogens, where studies are lacking in comparison to *P. aeruginosa*.
iii. The biochemical characterization of Zn transport systems. Many of these systems are only described by homology, but their involvement in the transport of specific metals has not yet been clearly demonstrated.

These research directions could open the way to a renewed and more integrated understanding of metal homeostasis in the genus *Pseudomonas*.
